# Lipid metabolism reprogramming and its potential targets in cancer

**DOI:** 10.1186/s40880-018-0301-4

**Published:** 2018-05-21

**Authors:** Chunming Cheng, Feng Geng, Xiang Cheng, Deliang Guo

**Affiliations:** Department of Radiation Oncology, The Ohio State University James Comprehensive Cancer Center and College of Medicine, Columbus, OH 43210 USA

**Keywords:** Lipid metabolism, Cancer, SCAP, SREBPs, Fatty acids, Cholesterol, Lipid droplets

## Abstract

Reprogramming of lipid metabolism is a newly recognized hallmark of malignancy. Increased lipid uptake, storage and lipogenesis occur in a variety of cancers and contribute to rapid tumor growth. Lipids constitute the basic structure of membranes and also function as signaling molecules and energy sources. Sterol regulatory element-binding proteins (SREBPs), a family of membrane-bound transcription factors in the endoplasmic reticulum, play a central role in the regulation of lipid metabolism. Recent studies have revealed that SREBPs are highly up-regulated in various cancers and promote tumor growth. SREBP cleavage-activating protein is a key transporter in the trafficking and activation of SREBPs as well as a critical glucose sensor, thus linking glucose metabolism and de novo lipid synthesis. Targeting altered lipid metabolic pathways has become a promising anti-cancer strategy. This review summarizes recent progress in our understanding of lipid metabolism regulation in malignancy, and highlights potential molecular targets and their inhibitors for cancer treatment.

## Background

Lipids, also known as fats, comprise thousands of different types of molecules, including phospholipids, fatty acids, triglycerides, sphingolipids, cholesterol, and cholesteryl esters. Lipids are widely distributed in cellular organelles and are critical components of all membranes [1–6]. In addition to their role as structural components, lipids in membranes also serve important functions of different organelles. Lipids could function as second messengers to transduce signals within cells, and serve as important energy sources when nutrients are limited [7–10]. Dysregulation of lipid metabolism contributes to the progression of various metabolic diseases, including cardiovascular diseases, obesity, hepatic steatosis, and diabetes [11–16].

**Fig. 1:**
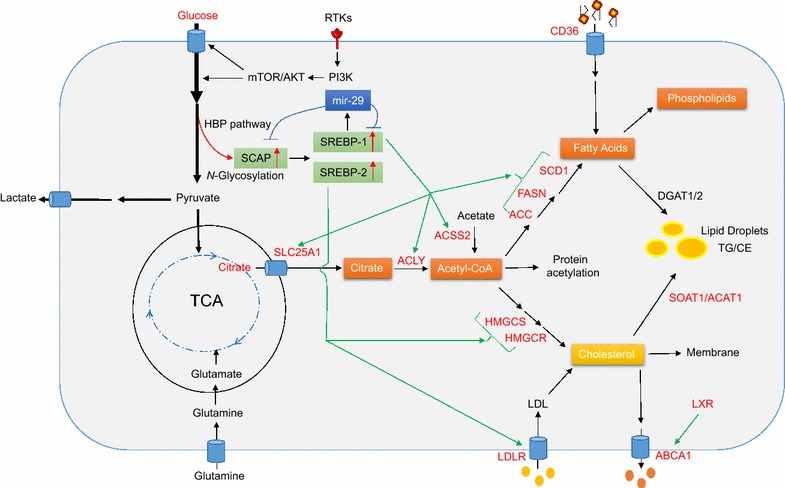
Regulation of lipid metabolism in cancer cells. In cancer cells, glucose uptake and glycolysis are markedly up-regulated by RTKs via the PI3K/Akt/mTOR signaling pathway, generating large amounts of pyruvate. Pyruvate is converted to lactate and it also enters the mitochondria, where it forms citrate, which is transported by SLC25A1 from the mitochondria into the cytoplasm, where the citrate serves as a precursor for de novo synthesis of fatty acids and cholesterol. Glutamine can also enter into mitochondria and participate in energy production and lipid synthesis. Acetate is converted to acetyl-CoA by the ACSS2 enzyme, serving as another source of lipid synthesis. Glucose participates in the HBP to form glycans that will be added to proteins during glycosylation. Oncogenic EGFR signaling increases *N*-glycosylation of SCAP, which activates SREBP-1 and -2 [55, 58], which ultimately up-regulate expression of enzymes in lipogenesis pathways and expression of LDLR. The enzyme up-regulation promotes fatty acid and cholesterol synthesis, while the LDLR up-regulation increases cholesterol uptake [40]. The microRNA miRNA-29 regulates the SCAP/SREBP pathway via a novel negative feedback loop [101]. The transporter CD36 brings fatty acids into cancer cells. When cellular fatty acids and cholesterol are in excess, they can be converted to TG and CE by the enzymes DGAT1/2 and SOAT1/ACAT1, forming LDs. When present in excess, cholesterol can be converted to 22- or 27-hydroxycholesterol, which activate LXR to up-regulate ABCA1 expression, promoting cholesterol efflux. *ABCA1* ATP-binding cassette transporters A, *ACC* acetyl-CoA carboxylase, *ACLY* ATP citrate lyase, *ACSS2* acetyl-CoA synthetase 2, *DGAT1/2* diacylglycerol *O*-acyltransferase 1/2, *FAs* fatty acids, *FASN* fatty acid synthase, *HBP* hexosamine biosynthesis pathway, *HMGCR* 3-hydroxy-3-methylglutaryl-CoA reductase, *HMGCS* 3-hydroxy-3-methylglutaryl-CoA synthase, *LD* lipid droplet, *LDLR* low-density lipoprotein receptor, *LXR* liver X receptor, *RTKs* oncogenic tyrosine kinase receptors, *SCAP* SREBP cleavage-activating protein, *SCD1* stearoyl-CoA desaturase 1, *SLC25A1* solute carrier family 25 member 1, *SOAT1 (also known as ACAT1)* sterol *O*-acyltransferase, *SREBPs* sterol regulatory element-binding proteins, *TG/CE* triglycerides/cholesteryl esters

Mammalian cells acquire lipids through two mechanisms, i.e., de novo synthesis and uptake. Accumulating evidence has demonstrated that lipid metabolism is substantially reprogrammed in cancers [17–22]. Lipogenesis is strongly up-regulated in human cancers to satisfy the demands of increased membrane biogenesis [7, 8, 21, 23]. Lipid uptake and storage are also elevated in malignant tumors [24–33]. Sterol regulatory element-binding proteins (SREBPs) are key transcription factors that regulate the expression of genes involved in lipid synthesis and uptake, and play a central role in lipid metabolism under both physiological and pathological conditions (Fig. 1). Dysregulation of SREBPs occurs in various metabolic syndromes and cancers [34–46]. Targeting the pathways regulating lipid metabolism has become a novel anti-cancer strategy. In this review, we summarize the recent progress in lipid metabolic regulation in malignancies, and discuss molecular targets for novel cancer therapy.

### Nutrient sources for lipid synthesis

Glucose is the major substrate for de novo lipid synthesis (Fig. 1). It is converted to pyruvate through glycolysis, and enters mitochondria to form citrate, which is then released into the cytoplasm to serve as a precursor for the synthesis of both fatty acids and cholesterol [47, 48]. Multiple glucose transporters as well as a series of enzymes that regulate glycolysis and lipid synthesis are strongly up-regulated in cancer cells [20, 21, 28, 49–54]. Glucose also participates in the hexosamine biosynthesis pathway to generate essential metabolites for the glycosylation of numerous proteins and lipids [55–57]. In this way, glycosylation is linked to the regulation of lipid metabolism [55, 58].

Glutamine could also be used for energy production and lipid synthesis via the tricarboxylic acid cycle in mitochondria [59–62]. Glutamine is the most abundant amino acid in the blood and tissues [63, 64]. It is a major nitrogen donor essential for tumor growth. Glutamine transporters, such as SLC1A5 (also known as ASCT2), are up-regulated in various cancers [65, 66]. After entering cells, glutamine can be converted to glutamate and α-ketoglutarate in the mitochondria, and generate ATP through oxidative phosphorylation [59–61, 67, 68]. Under conditions of hypoxia or defective mitochondria, glutamine-derived α-ketoglutarate is converted to citrate through reductive carboxylation and thereby contributes to de novo lipid synthesis [34, 69–71]. Acetate can also serve as a substrate for lipid synthesis after it is converted to acetyl-CoA in the cytoplasm [72–74].

### De novo lipid synthesis

Key regulators of lipogenesis—SREBPs, acetyl-CoA carboxylase (ACC), fatty acid synthase (FASN), and stearoyl-CoA desaturase 1 (SCD1) [27, 75–81]—are significantly up-regulated in various human cancers [20, 21, 28, 49–51]. Below we detail the roles of these proteins and discuss their potential as molecular targets in cancer treatment.

#### SCAP/SREBPs

SREBPs are a family of basic-helix-loop-helix leucine zipper transcription factors that regulate de novo synthesis of fatty acids and cholesterol as well as cholesterol uptake [11, 12, 82]. Mammalian cells express three SREBP proteins, SREBP-1a, -1c and -2, which are encoded by two genes, *SREBF1* and *SREBF2*. *SREBF1* encodes SREBP-1a and -1c proteins via alternative transcriptional start sites. The SREBP-1a protein is ~ 24 amino acids longer than -1c at its NH_2_-terminus, and has stronger transcriptional activity. SREBP-1a regulates fatty acid and cholesterol synthesis as well as cholesterol uptake, whereas SREBP-1c mainly controls fatty acid synthesis [83–86]. *SREBF2* encodes the SREBP-2 protein, and plays a major role in the regulation of cholesterol synthesis and uptake [87–92].

SREBPs are synthesized as inactive precursors that interact with SREBP cleavage-activating protein (SCAP), a polytopic transmembrane protein that binds to the insulin-induced gene protein (Insig), which is anchored to the endoplasmic reticulum (ER). The resulting Insig/SCAP/SREBP complex is retained in the ER [93–95]. Dissociation of SCAP from Insig, followed by a conformational change in SCAP, activates SREBP transcriptional activity. Conformational change in SCAP exposes a specific motif that allows SCAP to bind to Sec23/24 proteins, generating COPII-mediated translocation vesicles. SCAP mediates the entry of SREBPs into COPII vesicles that transport the SCAP/SREBP complex from the ER to the Golgi. In the Golgi, site 1 and 2 proteases (S1P and S2P) sequentially cleave SREBPs to release their N-terminal domains, which enter the nucleus and activate the transcription of genes involved in lipid synthesis and uptake (Fig. 1) [11, 12, 87, 88, 95, 96]. This process is negatively regulated by ER sterols, which are able to bind to SCAP or Insig and enhance their association, leading to the retention of SCAP/SREBP in the ER and reduction of SREBP activation [97–100]. Our research group recently showed that microRNA-29 (miR-29) participates in the negative feedback control of the SCAP/SREBP signaling pathway. We found that SREBP-1 up-regulates miR-29 transcription, and the microRNA binds to the 3′-untranslated region of SCAP and SREBP-1 transcripts and inhibit their translation [101, 102].

#### SCAP *N*-glycosylation

**Fig. 2:**
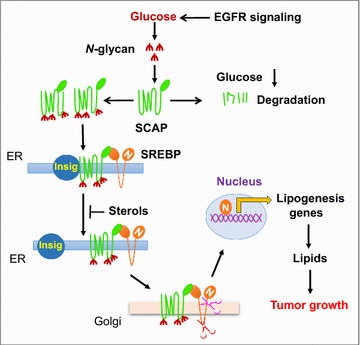
SCAP *N*-glycosylation is essential for SREBP trafficking and activation. SREBP activation is repressed by the ER-resident protein Insig, which binds to SCAP to prevent SREBP translocation and nuclear activation. The Nobel Prize-winning laboratories of Brown and Goldstein revealed that sterols modulate Insig interaction with SCAP to retain the SCAP/SREBP complex in the ER and inhibit SREBP [273, 274]. Our recent work has shown that glucose-mediated *N*-glycosylation stabilizes SCAP and promotes its dissociation from Insig, triggering the trafficking of the SCAP/SREBP complex from the ER to the Golgi, where SREBPs are cleaved to release their transcriptionally active N-terminal fragments to activate lipogenesis for tumor growth [55]. We further showed that EGFR signaling enhances glucose intake and thereby promotes SCAP *N*-glycosylation and SREBP activation

A recent series of studies in our laboratory showed that glucose could activate SCAP/SREBP trafficking and activation (Fig. 2) [55, 103, 104]. We tested the effects of glucose intermediate metabolites on different metabolic pathways, including glycolysis, oxidative phosphorylation, and hexosamine synthesis for glycosylation. We found that only *N*-acetylglucosamine (GlcNAc), an intermediate in the hexosamine biosynthesis pathway, activates SREBPs when glucose supply is limited. We found that inhibiting *N*-glycosylation, but not *O*-glycosylation, abolished glucose-mediated SCAP up-regulation and SREBP activation, indicating that glucose-mediated *N*-glycosylation of SCAP is essential for SCAP/SREBP trafficking and activation. These findings also demonstrated a coordinated molecular regulation mechanism that links glucose availability and the rate of de novo lipid synthesis (Fig. 2) [55, 58, 105].

#### SREBP activation in malignancy

The importance of SREBPs in cancer has begun to be recognized. Our group discovered that SREBP-1 is markedly up-regulated in glioblastoma [34, 106–108], the most common primary brain tumor and one of the most lethal cancers [34, 109–113]. Glioblastomas depend strongly on lipogenesis for rapid growth when they express the amplified tyrosine kinase receptor called epidermal growth factor receptor (EGFR) or its constitutively active mutant form EGFRvIII. This mutant lacks a portion of the extracellular ligand-binding domain [34, 106, 108, 111, 114, 115]. EGFR/EGFRvIII promotes lipid synthesis by activating SREBP-1 via PI3K/Akt signaling [12, 34, 87]. The nuclei of human glioblastoma cells display elevated levels of SREBP-1 [34], suggesting that the SCAP/SREBP complex may escape the tight repression of Insig, leading to high SREBP activation. Other groups have found elevated SREBP-1 in various cancers, and SREBP-1 levels in various cell lines are regulated by PI3K/Akt signaling and mTORC1 [116–122]. How SREBP-1 is activated in cancer cells is not entirely understood and requires further investigation.

Inhibiting SREBPs at the genetic level or with pharmacological agents significantly suppresses tumor growth and induces cancer cell death, making SREBPs promising therapeutic targets [28, 34, 123–137]. However, directly inhibiting SREBPs is challenging, as transcription factors often make poor drug targets. A more promising approach is to inhibit SREBP translocation from the ER to the Golgi. Along this line, fatostatin, betulin and PF-429242 have been shown to inhibit SREBP activation and have promising anti-tumor effects in pre-clinical studies [126–131].

SREBP-2 is up-regulated in prostate cancer [37, 138]. SREBP-2 regulates 3-hydroxy-3-methylglutaryl-CoA (HMG-CoA) reductase, the rate-limiting enzyme for cholesterol synthesis. Inhibiting SREBP-2 has been explored as an anti-cancer therapy [139–144]. Statins are inhibitors of HMG-CoA reductase and are widely used to reduce circulating cholesterol levels. The anti-cancer effects of statins have been tested for various types of cancers, both pre-clinically and in patients [140, 142, 143, 145]. However, inhibition of cholesterol synthesis can lead to feedback activation of SREBPs, making the anti-cancer effects of statins less effective [144]. Thus, combination therapies that simultaneously inhibit cholesterol synthesis and SREBP activation are being developed [146, 147].

#### SLC25A1

A critical step for glucose-mediated de novo lipid synthesis is the release of citrate from mitochondria into the cytoplasm. Solute carrier family 25 member 1 (SLC25A1), also referred to as citrate carrier (CIC), functions as a key transporter to export citrate from mitochondria to the cytoplasm, providing a key precursor for both fatty acid and cholesterol synthesis [148, 149] (Fig. 1). SLC25A1 is regulated by SREBP-1 [150] and plays an important role in inflammation and tumor growth [151, 152]. In lung cancer cells, SLC25A1 is up-regulated by mutant p53 [151]. These findings, though preliminary, suggest that specific inhibitors of SLC25A1 may have anti-tumor effects.

#### ACLY

ATP citrate lyase (ACLY) converts cytoplasmic citrate to acetyl-CoA, a precursor of lipid synthesis (Fig. 1) [153–155] and a substrate for protein acetylation [153]. ACLY is a downstream target of SREBPs [156–158], and is up-regulated in many cancers, including glioblastoma, colorectal cancer, breast cancer, non-small cell lung cancer, and hepatocellular carcinoma [159–161]. Inhibiting ACLY at the genetic level or pharmacologically significantly suppresses tumor growth [162–164]. The ACLY inhibitor SB-204990 strongly inhibits tumor growth in mice with lung, prostate or ovarian cancer xenografts [162, 165]. These results suggest that ACLY may serve as an attractive anti-cancer target [155].

#### ACSS2

Acetate is converted to acetyl-CoA by acetyl-CoA synthetases (ACSSs), making acetate an important molecule for lipid synthesis and histone acetylation [7]. In mammalian cells, ACSS isoforms 1 and 3 localize to the mitochondria, whereas isoform 2 is found in the cytoplasm and nucleus [166]. Isoform 2 expression is regulated by SREBPs [167]. When each isoform was genetically knocked down in HepG2 cells, only ACSS2 down-regulation dramatically suppressed acetate-mediated lipid synthesis and histone modification [72]. In fact, ACSS2 expression correlates inversely with overall survival in patients with triple-negative breast cancer, liver cancer, glioma or lung cancer [72, 73, 168, 169]. Studies with patient-derived glioblastoma xenografts have shown that acetate contributes to acetyl-CoA synthesis in tumors [73]. Indeed, cancer cells rely mainly on acetate as a carbon source for fatty acid synthesis under hypoxic conditions [74]. Knocking down ACSS2 suppresses proliferation of several cancer cell lines as well as growth of xenograft tumors [74, 170–173]. ACSS2 also participates in autophagy when glucose supply is limited: it triggers histone acetylation in the promoter regions of autophagy genes, enhancing their expression [174, 175].

#### ACCs

Following the conversion of citrate and acetate to acetyl-CoA, the ACC enzymes catalyze ATP-dependent carboxylation of acetyl-CoA, generating malonyl-CoA for fatty acid synthesis (Fig. 1). Two ACC isoforms have been identified in mammalian cells, ACC-alpha (also termed ACC1) and ACC-beta (also known as ACC2) [176, 177]. ACC is up-regulated in several human cancers, including glioblastoma and head and neck squamous cell carcinoma [34, 178]. Inhibiting ACCs significantly reduces fatty acid synthesis and suppresses tumor growth in various xenograft models [179–186]. The ACC inhibitors TOFA, soraphen A and ND646 have shown significant anti-tumor effects in xenograft tumor models (Table 1) [179–184].

**Table 1:** Representative targets within the lipid metabolism pathway for anti-cancer drug development

Target protein	Inhibitor	Type of cancer	Preclinical model	Clinical trial	References
SCAP	–	GBM	Xenografts	–	[55]
SREBPs	Fatostatin, betulin, PF-429242, xanthohumol	GBM, prostate, liver, skin, melanoma, colorectal, bile duct, pancreatic, and breast cancer	Xenografts	–	[28, 125–138]
ACCs	TOFA, soraphen A, ND-646	Lung, ovarian cancer, head and neck squamous cell carcinoma	Xenografts	–	[179–185]
ACLY	SB-204990, bempedoic acid, BMS303141	Lung, prostate, and ovarian cancer	Xenografts	–	[152, 162, 165]
FASN	Cerulenin	Ovarian cancer, breast cancer	Xenografts	–	[179, 194–196]
	C75	Breast, GBM, renal, and mesothelioma cancer	Xenografts	–	[34, 179, 188, 197–203]
	TVB-2640	Solid malignant tumors	–	Phase I	Clinicaltrials.gov (NCT02223247), [191]
	TVB-3166	Lung, ovary, and pancreatic cancer	Xenografts	–	[264]
	C93	Ovarian and lung cancer	Xenografts	–	[265, 266]
	C247	Breast cancer		–	[267]
	Orlistat	Prostate cancer and melanoma	Xenografts	–	[192, 193]
	Triclosan	Breast cancer	Xenografts	–	[268, 269]
LDLR	–	GBM	–	–	[27, 219]
SCD1	BZ36, A939572, MF-438	Prostate, renal cancer	Xenografts	–	[124, 212–215, 270]
LXR	GW3965, LXR-623	GBM	Xenografts	–	[27, 238, 239]
	SR9243	Prostate cancer	Xenografts		[242]
SOAT1 (or ACAT1)	K604, ATR-101, avasimibe	GBM, prostate and pancreatic cancer	Xenografts	–	[28, 230–232]
CPT1	Etomoxir, perhexiline	Leukemia, prostate and breast cancer	Xenografts, transgenic mice	–	[248–250, 271, 272]
CD36	Anti-CD36 antibodies	Oral cancer	Xenografts	–	[24–26]

*ACCs* acetyl-CoA carboxylases, *ACLY* ATP citrate lyase, *CD36* cluster of differentiation 36, also known as fatty acid translocase (FAT), *CPT1* carnitine palmitoyltransferase 1, *FASN* fatty acid synthase, *GBM* glioblastoma multiforme, *LDLR* low-density lipoprotein receptor, *LXR* liver X receptor, *SCAP* SREBP cleavage-activating protein, *SREBPs* sterol regulatory element-binding proteins

#### FASN

Fatty acid synthase (FASN), a key lipogenic enzyme catalyzing the last step in de novo biogenesis of fatty acids, has been studied extensively in various cancers [21, 187–191]. The early-generation FASN inhibitors C75, cerulenin and orlistat (Table 1) have been studied pre-clinically, but their pharmacology and side effects limited their potential for clinical use [34, 179, 188–203]. The later-generation inhibitor TVB-2640 has entered clinical trials in patients with solid tumors (Table 1) [21, 191, 204, 205].

#### SCD1

Stearoyl-CoA desaturase (SCD) is an ER-resident integral membrane protein that catalyzes the formation of the mono-unsaturated fatty acids oleic acid (18:1) or palmitoleic acid (16:1) from stearoyl-(18:0) or palmitoyl-CoA (16:0) [206, 207]. There are 5 *SCD* genes (*SCD1*-*5*). Humans contain the *SCD* homologs *SCD1* and *SCD5*, but the function of SCD5 remains unknown [208–210]. The mono-unsaturated products of SCD1 are key substrates in the formation of membrane phospholipids, cholesteryl esters and triglycerides, making SCD1 a promising anti-cancer target [75, 211]. The SCD1 inhibitors BZ36, A939572 and MF-438 have shown anti-tumor effects in pre-clinical xenograft models (Table 1) [212–215].

### Lipid uptake

#### CD36

In addition to de novo synthesis, lipid uptake from the exogenous environment is another important route through which cells acquire fatty acids. CD36 transports fatty acids into the cell [216, 217], and plays a critical role in cancer cell growth, metastasis and the epithelial-mesenchymal transition [24–26]. An anti-CD36 antibody has shown significant anti-metastatic efficacy in oral cancer xenograft models [25].

#### LDLR

Cholesterol is an essential structural component of cell membranes [2, 218]. Cholesterol could be synthesized by cells de novo or through internalizing low-density lipoprotein (LDL). LDL binds to the membrane-bound LDL receptor (LDLR) and is internalized, after which it enters lysosomes, where free cholesterol is released [11, 76]. LDLR is up-regulated in glioblastoma via EGFR/PI3K/Akt/SREBP-1 signaling [27], and plays an important role in tumor growth [27, 76, 219]. LDLR has not been investigated as an anti-cancer target.

### Lipid storage/lipid droplets

#### SOAT1/ACAT1

When cellular lipids are in excess, they are converted to triglycerides and cholesteryl esters in the ER, forming lipid droplets [220–222]. These droplets have been observed in various types of tumor, including glioblastoma, renal clear cell carcinoma, and cancers of the prostate, colon or pancreas [29–33]. Diglyceride acyltransferase 1/2 (DGAT1/2) could synthesize triglyceride from diacylglycerol and acyl-CoA (Fig. 1) [223, 224]. So far, the role of triglycerides in cancer cells has not been explored.

Cholesteryl esters are abundant in tumor tissue, while they are usually undetectable in normal tissue [225–229]. Sterol *O*-acyltransferase 1 (SOAT1), also known as acyl-CoA acyltransferase 1 (ACAT1), converts cholesterol to cholesteryl esters for storage in lipid droplets (Fig. 1). This enzyme is highly expressed in glioblastomas and in cancer of the prostate or pancreas; its expression level correlates inversely with patient survival [28, 29, 230–235]. Genetically silencing SOAT1/ACAT1 or blocking its activity using the inhibitors K604, ATR-101 or avasimibe effectively suppresses tumor growth in several cancer xenograft models [28, 230–232]. These results suggest that targeting SOAT1 and cholesteryl ester synthesis may be a promising anti-cancer strategy.

### Cholesterol efflux

#### LXR/ABCA1

Cholesterol homeostasis is critical for maintaining cellular function, and is regulated by de novo synthesis, uptake, storage, and efflux [11, 76]. Increases in cholesterol levels can trigger feedback inhibition of cholesterol biosynthesis or conversion of cholesterol into cholesteryl esters stored in lipid droplets. Levels of 22- or 27-hydroxycholesterol can also increase, and these molecules bind to and activate the liver X receptor, which turns on expression of ATP-binding cassette proteins A1 (ABCA1) and G1 (ABCG1) [236]. Both proteins are plasma membrane-bound transporters that promote cholesterol export and thereby reduce intracellular cholesterol levels [237]. Synthetic liver X receptor agonists GW3965 and T0901317 significantly inhibit tumor growth in animal models of glioblastoma, breast cancer or prostate cancer [7, 27]. Activation of the liver X receptor by GW3965 up-regulates a ubiquitin ligase E3 that degrades LDLR [27, 62, 238]. The highly brain-penetrant liver X receptor agonist LXR-623 selectively kills glioblastoma cells and prolongs survival of glioblastoma-bearing mice [239]. Therefore, the combination of increasing cholesterol efflux by activating the liver X receptor and decreasing cholesterol uptake may be a promising anti-cancer strategy.

Activation of liver X receptor up-regulates transcription of glycolysis genes, such as those encoding PFK2 and GCK1, as well as of lipogenesis genes, such as those encoding SREBP-1c, FASN, and SCD [240, 241]. Conversely, inhibiting the liver X receptor using the inverse agonist SR9243 downregulates expression of PFK2 and SREBP-1c, thereby inhibiting glycolysis and fatty acid synthesis as well as suppressing xenograft tumor growth [242]. These results suggest that developing antagonists against liver X receptors may be a new anti-cancer direction. However, such an approach can be effective only if the liver X receptor shows high transcriptional activity in human tumors, which has not been clearly demonstrated yet. Moreover, inhibiting liver X receptors alone may be insufficient for reducing glycolysis and lipogenesis in human tumors, since these metabolic programs are up-regulated by multiple oncogenic signaling pathways [243–245]. Regardless, efforts to inhibit cancer growth by using liver X receptor agonists to activate cholesterol efflux can be undermined by the concomitant up-regulation of glycolysis and lipogenesis. It may be more effective to simultaneously enhance cholesterol efflux and inhibit glycolysis and lipogenesis.

### Fatty acid oxidation

#### CPT1

Fatty acids are an important energy source for cell growth and survival when nutrients are limiting. Carnitine palmitoyltransferase I (CPT1) converts fatty acids to acylcarnitines, which are shuttled into mitochondria, where they undergo β-oxidation and produce energy [21]. Fatty acid β-oxidation plays a critical role in tumor growth [246, 247], and the CPT1 inhibitors etomoxir and perhexiline have been tested for anti-cancer effects in various animal models [248–250].

### Lipid peroxidation and cell death

Lipids, particularly polyunsaturated fatty acids, are susceptible to oxidation by oxygen free radicals, leading to lipid peroxidation that is harmful to cells and tissues [251–253]. Lipid peroxides are associated with many pathological states, including inflammation, neurodegenerative disease, cancer, and ocular and kidney degeneration [253, 254]. Lipid peroxidation triggers the propagation of lipid reactive oxygen species that can significantly alter the physical properties of cellular membranes, or degrade into reactive compounds that cross-link DNA or proteins, exerting further toxic effects [253, 255, 256]. Extensive lipid peroxidation can result in ferroptosis, a regulated form of iron-dependent, non-apoptotic cell death [255, 257]. Inducing ferroptosis may be an anti-cancer strategy [257–259]. For example, disrupting the repair of oxidative damage to bio-membranes by inhibiting the antioxidant enzyme glutathione peroxidase 4 (GPX4) could induce ferroptosis [257, 259–262]. This has emerged as an active area of research that may lead to new anti-cancer approaches, particularly against metabolically active tumors.

### Summary

Extensive studies have provided strong evidence for reprogramming of lipid metabolism in cancer [27, 34, 55]. A variety of lipid synthesis inhibitors have shown promising anti-cancer effects in preclinical studies and early phases of clinical trials [7, 29, 55, 263]. However, major barriers exists in developing cancer treatment by targeting altered lipid metabolism, mostly due to incomplete understanding of the mechanisms that regulate lipid synthesis, storage, utilization and efflux in cancer cells.
